# Formation and Evaluation of Silicon Substrate with Highly-Doped Porous Si Layers Formed by Metal-Assisted Chemical Etching

**DOI:** 10.1186/s11671-021-03524-z

**Published:** 2021-04-20

**Authors:** Yijie Li, Nguyen Van Toan, Zhuqing Wang, Khairul Fadzli Bin Samat, Takahito Ono

**Affiliations:** 1 Department of Mechanical Systems Engineering, Tohoku University, Aoba-ku Sendai, 980-8579 Japan; 2 Micro System Integration Center, Tohoku University, Aoba-ku Sendai, 980-8579 Japan; 3 Fakulti Kejuruteraan Pembuatan, Universiti Teknikal Malaysia Melaka, 76100 Durian Tunggal, Melaka Malaysia

**Keywords:** Porous Si, Metal-assisted chemical etching, Spin on dopant, Thermoelectric, Power factor

## Abstract

Porous silicon (Si) is a low thermal conductivity material, which has high potential for thermoelectric devices. However, low output performance of porous Si hinders the development of thermoelectric performance due to low electrical conductivity. The large contact resistance from nonlinear contact between porous Si and metal is one reason for the reduction of electrical conductivity. In this paper, *p*- and *n*-type porous Si were formed on Si substrate by metal-assisted chemical etching. To decrease contact resistance, *p*- and *n*-type spin on dopants are employed to dope an impurity element into *p*- and *n*-type porous Si surface, respectively. Compared to the Si substrate with undoped porous samples, ohmic contact can be obtained, and the electrical conductivity of doped *p*- and *n*-type porous Si can be improved to 1160 and 1390 S/m, respectively. Compared with the Si substrate, the special contact resistances for the doped *p*- and *n*-type porous Si layer decreases to 1.35 and 1.16 mΩ/cm^2^, respectively, by increasing the carrier concentration. However, the increase of the carrier concentration induces the decline of the Seebeck coefficient for *p*- and *n*-type Si substrates with doped porous Si samples to 491 and 480 μV/K, respectively. Power factor is related to the Seebeck coefficient and electrical conductivity of thermoelectric material, which is one vital factor that evaluates its output performance. Therefore, even though the Seebeck coefficient values of Si substrates with doped porous Si samples decrease, the doped porous Si layer can improve the power factor compared to undoped samples due to the enhancement of electrical conductivity, which facilitates its development for thermoelectric application.

## Introduction

Nowadays, thermoelectric energy conversion, which can convert a wasted heat into an electrical energy, is regarded as an important technology to alleviate the pressure of energy shortage by increasing the energy utilization efficiency [1]. Especially, various sensor applications are highly demanded for future information communication society [2, 3]. Microfabrication technologies based on thermoelectric materials have been studied to realize highly efficient and compact thermoelectric devices [4–6]. The thermoelectric performance of thermoelectric materials is evaluated by the figure of merit *ZT* = *бS*^*2*^*T/к*, where *б* is electrical conductivity, *S* Seebeck coefficient, *T* absolute temperature, and *к* thermal conductivity [7]. The power factor, *бS*^*2*^, is the maximum generated power output of a material and thus is used to estimate its output performance.

Common thermoelectric materials, such as Bi_2_Te_3_ and Sb_2_Te_3_, contain rare and toxic materials, which make the large-scale production difficult. Si is considered as one of alternative candidates to apply to thermoelectric devices because it is abundant and widely used in semiconductor industry [8]. However, Si is not ideal thermoelectrical material due to its high thermal conductivity (~ 150 W/mK) and low *ZT* value (0.006) [9]. Recently, nanostructured Si has been proved to decrease the thermal conductivity to 1.6 W/mK and then improve the *ZT* value to 0.6 at 300 K [10]. Thermoelectrical generators based on Si nanowires are successfully fabricated recently [11, 12]. However, the performance of those thermoelectric generators is still low because the Si nanowire arrays are hard to guarantee the uniform diameter and smooth surface, which are strongly related to the *ZT* value.

Porous Si, as one of Si nanostructures, is regarded as one of candidates for thermoelectrical materials from theoretical and experimental works [13–15]. However, inadequate output performance of porous Si limits its development of thermoelectrical devices due to its low electrical conductivity. Many works found that the electrical contact between a metal and the porous Si shows nonlinear behavior due to the potential energy barrier between metal and porous Si [16, 17]. The reason is that the large surface states on porous Si and surface trapping effect cause the depletion of the carriers concentration on the surface of the porous Si [18]. It is a challenge for fabricating thermoelectrical generators because a large parasitic loss arises from the electrical resistance at the interface [19]. In addition, this increase of the barrier also decreases the apparent electrical conductivity of porous Si to several orders of magnitude compared to that of bulk Si, resulting in low power factor [20].

Impurity doping using a spin on dopant (SOD) is one surface modification method to increase the electrical conductivity by increasing the carrier concentration on Si surface. Unlike ion implantation, impurity doping with SOD is less damage process [21]. Some works have indicated that a Si nanowire doped by SOD exhibits a high electrical conductivity [22, 23]. Boor et al. found that the electrical conductivity of a porous Si film formed by the electrochemical method can be enhanced after doping with SOD [24]. However, the electrical contact characteristic of porous Si doped with SOD is not investigated in previous studies and is an important parameter for thermoelectric devices. Moreover, the investigation of SOD doping effect on the output performance of Si substrate with porous Si layer is rare. Different from the electrochemical method, we used metal-assisted chemical etching (MACE) to form porous Si. MACE is a simple and mass-producible method using a wet-etching technique, which can synthesize diverse nanostructures including Si nanowire and porous Si [25–27]. Moreover, it can form straight nanopores at faster etching rate than that of other methods.

In this study, *p*- and *n*-type Si substrate with porous Si layers were formed by MACE, and the Seebeck coefficient, electrical conductivity and electrical contact were evaluated on the porous Si layers doped with different types of SOD. First, the Seebeck coefficient of *p*- and *n-*type porous Si formed by MACE were investigated and the carrier concentration of the doped layer with SOD was evaluated to verify the doping effect. Then, the electrical conductivities and contact characteristics of Si substrates with doped porous Si layers were measured using current–voltage characteristics. Moreover, the electrical contact between metal and doped porous Si layer was evaluated in terms of special contact resistance. Finally, the power factors of *p*- and *n*-type Si substrates with doped porous Si layers were evaluated and compared with undoped samples.

## Methods

**Fig. 1:**
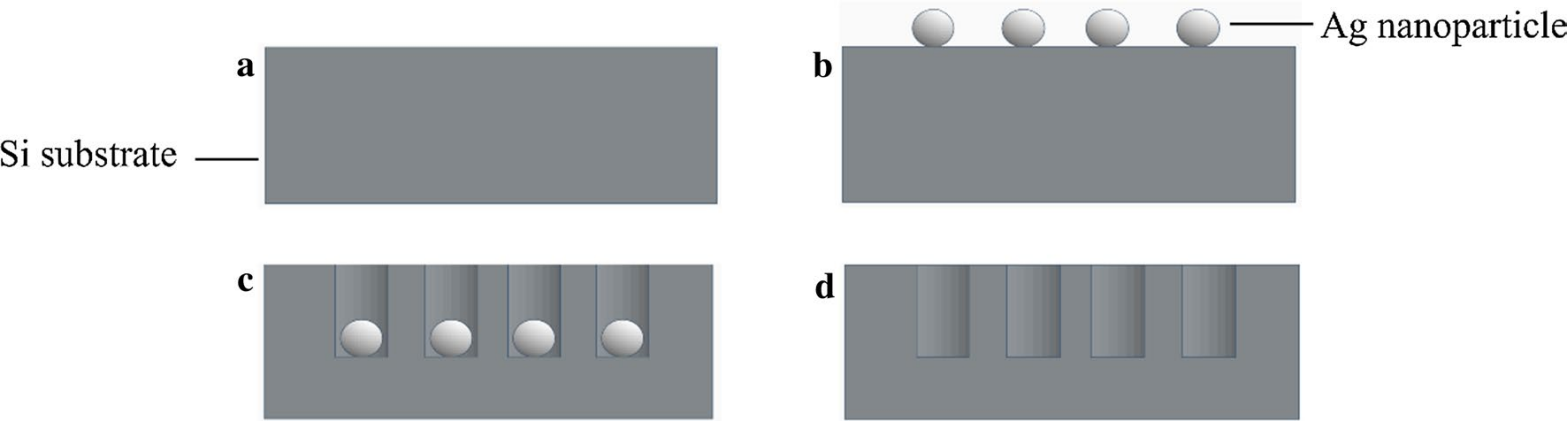
Si substrate with porous Si layer formation process using metal-assisted chemical etching. **a** Cleaned Si substrate. **b** Ag nanoparticle deposition. **c** etching with a HF solution. **d** Silver nanoparticles removal

(100)-oriented *p*-type (10.0 mΩ-cm) and *n*-type (10.0 mΩ-cm) Si substrates with a size of 2 × 2 cm^2^ were sequentially cleaned in acetone, ethanol, deionized water and piranha solution (H_2_SO_4_:H_2_O_2_ in the volume ratio of 2:1). Then, the *p*- and *n*-type porous Si layers were formed on the Si substrates by MACE process as follows. First, Si substrates were immersed in a mixture of 0.14 M HF and 5 × 10^–4^ M AgNO_3_ solutions for 7 min at room temperature. In this procedure, Ag nanoparticles, with a diameter ranging from 80 to 180 nm, were deposited on the Si surface, as shown in Fig. 1. Next, Si substrates deposited with Ag nanoparticles were immersed in an etching solution containing of 25 ml of 49% HF solution, 10 ml of 35% H_2_O_2_ solution, and 5 ml of deionized water in a volume ratio of 5:2:1 at room temperature. The etching reaction proceeds with electrical local anodization and oxide removal processes. The Ag nanoparticles immediately oxidize the Si surface at the interface by local anodization, and the oxide is etched by HF in the solution. As the etching proceeds, the Ag nanoparticles penetrate into Si for further etching. Hence, a porous Si layer is formed. It is reported that the porous formation rate for *p*-type Si is slower than that for *n*-type Si [28]. The thickness of porous Si is controlled by etching time. In our experiments, the etching time of *p*-type Si is selected to be 2 min 40 s while that of *n*-type Si is processed for 2 min to obtain around 20 μm porous Si film. Finally, the Si substrates were cleaned by 10% nitric acid to remove the Ag nanoparticles followed by deionized water cleaning. The porosity of porous Si layer is defined as follows,

1 $${\rm{Porosity}} = \frac{{m_{1} - m_{2} }}{pv}$$

where *m*_1_ is the mass of the initial sample, *m*_*2*_ is the mass of the sample after formation of the porous Si layer, *p, v* is the density and volume of the original etched single-crystal Si layer. The morphologies of the porous Si film were observed by field emission scanning electron microscopes (FE-SEM).

**Fig. 2:**
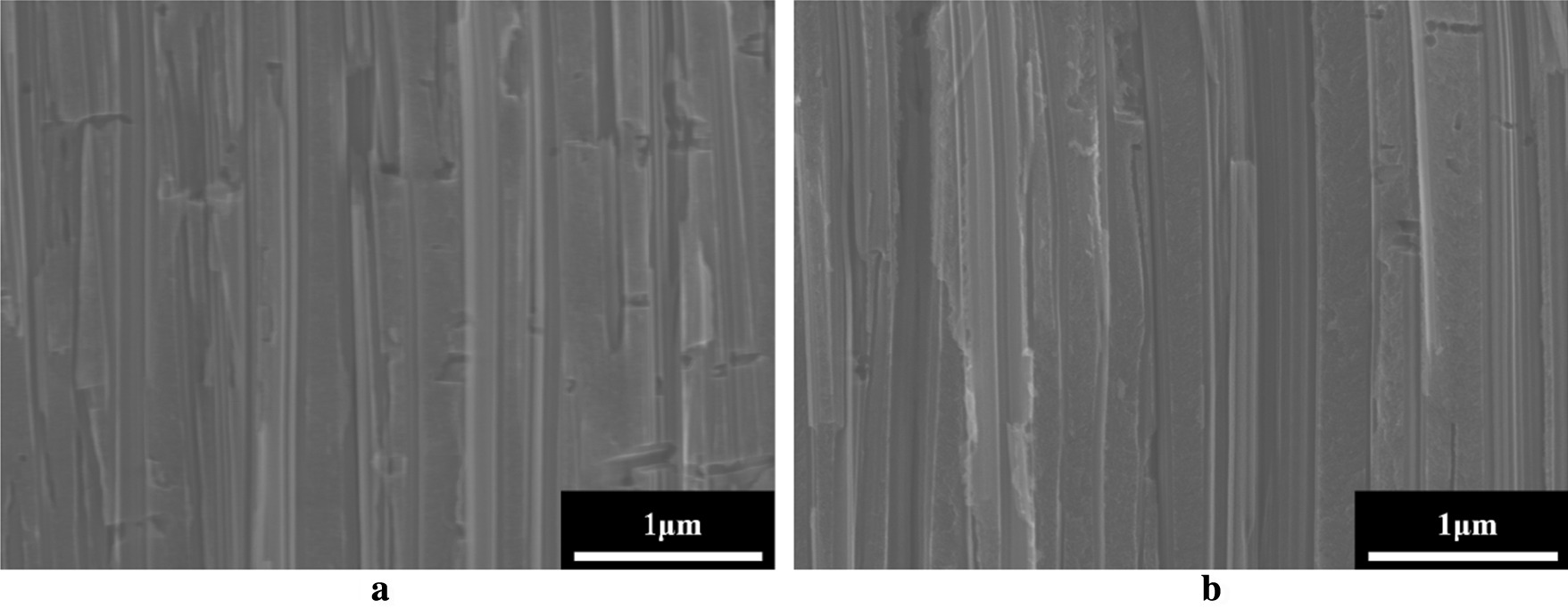
Microstructures of porous Si layers for **a** *p*-type and **b** *n*-type

Figure 2a, b depicts the cross-sectional images of *p*- and *n*-type porous Si films formed by MACE. The thickness of *p*- and *n*-type porous Si films is approximately 20 μm, which is adjusted by the etching time. The average pore diameters of *p*- and *n*-type porous Si films are 130 nm and 125 nm, respectively, and the porosities of *p*- and *n*-type porous Si films are 35% and 31%, respectively.

The impurities of boron or phosphorous were doped into the porous Si layer to form *p*-type or *n*-type Si layer, respectively, using a SOD (Filmtronics Inc. USA). SOD was coated on the surface by spin coating at 3000 rpm for 30 s. Then, the sample was baked at 110 °C for 15 min to harden the SOD film. Afterward, the samples were put into a quartz tube furnace and annealed at 1100 °C for 3 h in N_2_ environment to diffuse the dopant atoms into porous Si. Finally, the samples were immersed into a HF solution to remove SiO_2_ and clean up the surface. To validate the doping effect on the porous Si layer, the carrier concentration of undoped and doped porous Si layer was measured by a Hall Effect measurement system [17].

**Fig. 3:**
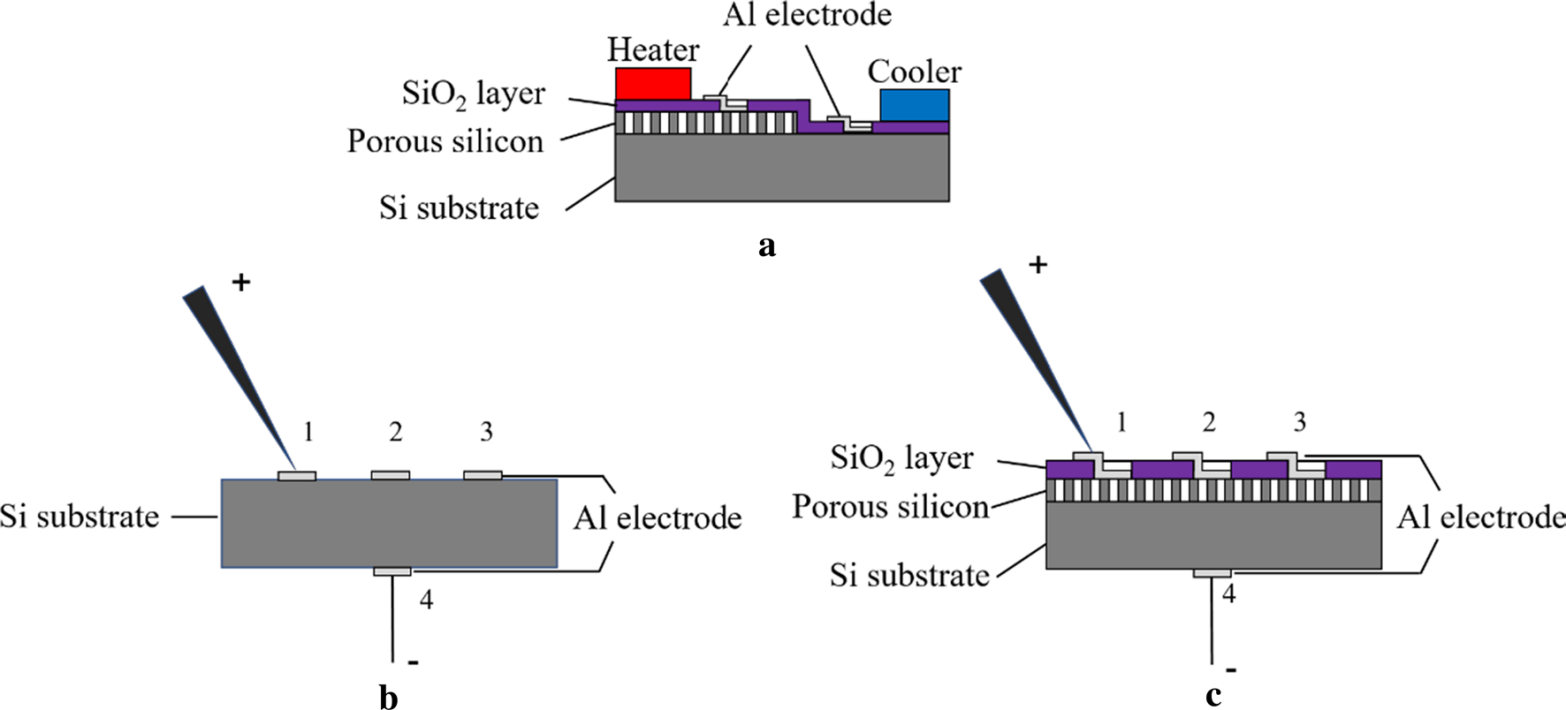
**a** Illustration of the device for the Seebeck coefficient measurement; illustration of the device for the electrical conductivity and special contact resistant measurement: **b** Si substrate; **c** Si substrate with porous Si layer

Two metal electrode patterns were separately formed on the porous Si film and the backside of the Si substrate for the measurement of the cross-plane Seebeck coefficient of the samples at room temperature, as shown in Fig. 3a. The porous Si layer was formed on the half area of the Si wafer, and the remained part of Si was etched by 30 μm in depth using deep reactive ion etching (RIE). Then, 1-μm-thick SiO_2_ film was deposited on the surface by tetraethoxysilane chemical vapor deposition (TEOS-CVD) to decrease the heat loss to ambient atmosphere. Two 1 × 1 mm^2^ square contact windows were formed on the SiO_2_ film. After that, two 1 × 2 mm^2^-rectangular 300-nm-thick Ti-Au electrodes were formed by electron beam evaporation for contact pads. Finally, two commercial Peltier elements were contacted with the Si substrate for creating temperature gradient along in-plane direction. The temperatures *T*_*1*_ and *T*_*2*_ at two electrodes were measured by thermocouples and the difference of temperature Δ*T* were obtained. The generated voltage Δ*V* was measured by an electrometer. The Seebeck coefficient of the sample was obtained from equation below:

2 $$S = - \frac{\Delta V}{{\Delta T}}$$

The current (*I*)–voltage (*V*) characteristics were measured for *p*-type bulk Si, *n*-type bulk Si, and doped/undoped porous Si using lateral and vertical measurement configurations, as shown in Fig. 3b, c. Three 200-nm-thick Al electrodes (‘1,’ ‘2,’ ‘3’) with a size of 0.6 × 0.6 mm^2^ were formed on the top side of bulk Si samples and also an Al electrode (‘4’) was formed on back side as well, as shown in Fig. 3b. In order to make the electrical contact a copper wire was glued on the electrode ‘4′ using a silver paste. The center-to-center distance between Al electrodes ‘12,’ ‘23,’ ‘13’ were 0.2, 0.3, 0.56 cm, respectively. The bulk Si samples were annealed at 450 ℃ for 30 min to confirm the ohmic contact between Al and Si. To measure special contact resistance, the lateral resistances among three electrodes were measured using a high-sensitive probe. To measure vertical electrical conductivity of the bulk Si substrate, the electrical conductance between Al electrodes ‘2’–‘4’ was measured. To measure the electrical properties of the porous Si samples with and without doping, a 2-μm-thick SiO_2_ layer was deposited on the porous Si film by tetraethyl orthosilicate CVD (TEOS-CVD) to avoid the mechanical damage to the porous Si layer from the electrical probe. Three 0.6 × 0.6 mm^2^ SiO_2_ windows were formed by etching the SiO_2_ layer partly using a buffered HF to make electrical contact. Then, three 1.0 × 0.6 mm^2^ Al electrodes were formed on the SiO_2_ layer together with the SiO_2_ windows, as shown in Fig. 3c. Thus, the probe can physically contact with the extended Al electrodes to make electric contact to the porous Si film.

## Results and Discussion

**Fig. 4:**
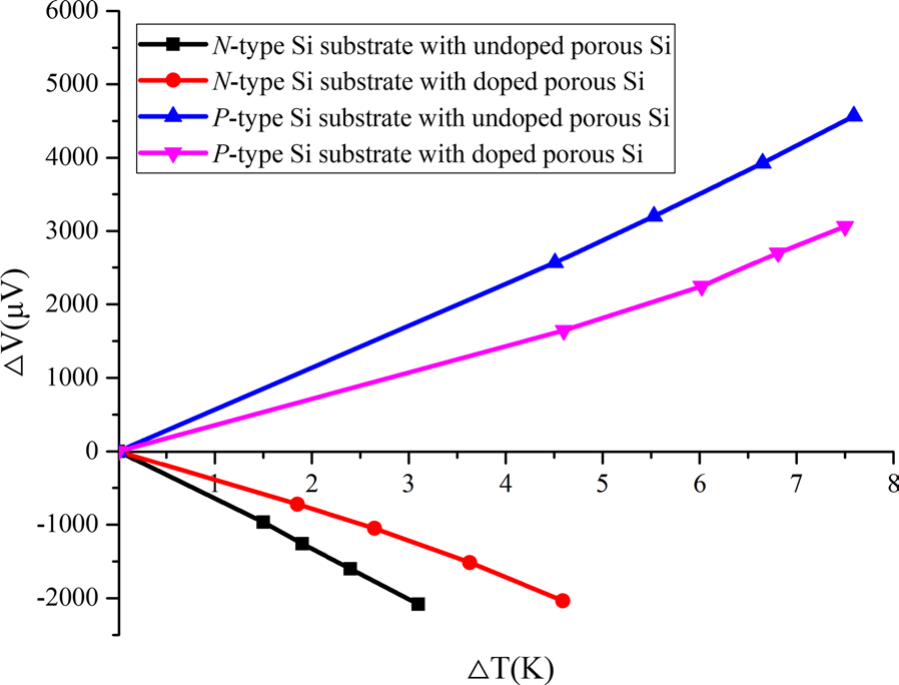
Generated voltage versus temperature difference on Si substrates with the doped and undoped n and p-type porous Si layers

Figure 4 shows that the generated voltage versus temperature difference on the Si substrate with doped and undoped porous Si samples. The calculated Seebeck coefficient values of different samples were shown in Table 1. The Seebeck coefficient for the bulk p and n-type Si are 450 and 485 μV/K, respectively, and the Seebeck coefficient for *p* and *n*-type Si substrates with the undoped porous Si sample shows higher values of 696 and 650 μV/K, respectively. This reason of the Seebeck coefficient difference is due to the energy filtering effect and surface scattering effect [29–31]. After the doping process, the Seebeck coefficient of *p* and *n* type Si substrates with the doped porous Si samples decreases to 491 and 480 μV/K because of the increase of the carrier concentration on the doped porous Si layers. However, for whole doped porous Si sample, the doped porous Si layer is thin and the remaining part of porous Si layer is still undoped. Therefore, even though the carrier concentration of doped porous Si layer is higher than that of bulk Si, the Seebeck coefficient of whole doped porous Si sample (doped porous Si layer + undoped porous Si layer) is close to that in the bulk Si samples.

**Table 1:** Seebeck coefficient, electrical conductivity, special contact resistance and power factor of *p* and *n*-type bulk Si and Si substrate with undoped and doped porous Si samples at room temperature

	Bulk *p*-Si	Bulk *n*-Si	Undoped *p*-porous Si	Undoped *n-*porous Si	Doped *p*-porous Si	Doped *n*-porous Si
Seebeck (μV/K)	450 ± 6	485 ± 8	696 ± 6	650 ± 7	491 ± 10	480 ± 10
Electrical conductivity (S/m)	10,000	10,000	150 ± 10	385 ± 12	1160 ± 3	1390 ± 3
Special contact resistance (mΩ-cm^2^)	1.88	1.25	–	–	1.35	1.16
Power factor [μW/(m K^2^)]	2025	2304	73	162	280	320

In general, the Seebeck coefficient is composed of the charge diffusion part *S*_*d*_ and phonon drag part *S*_*ph*_. Because all samples are highly doped (~ 10^18^) and the measurements are taken at room temperature, the *S*_*ph*_ value is far smaller than *S*_*d*_, resulting in *S* ≈ *S*_*d*_ [32]. Thus, the Seebeck coefficient can also be shown as fellows [33],

3 $$S = \frac{{8\pi^{2} k_{{\rm{B}}}^{2} T}}{{3qh^{2} }}m^{*} \left( {\frac{\pi }{3n}} \right)^{2}$$

where *k*_B_ is Boltzmann constant, *h* is Planck constant, *T* is absolute temperature, m* is state effective mass, *q* is the electron charge and *n* is the carrier concentration. Therefore, carrier concentration *n* is an important factor that determines the value of Seebeck coefficient.

To better understand the relationship between the carrier concentration and Seebeck coefficient, the carrier concentration of *p* and *n*-type doped and undoped porous Si layers was measured by Hall measurement. The carrier concentration of *p* and *n*-type undoped porous Si layers are found to be 1.3 × 10^18^ and 1.35 × 10^18^ cm^−3^, respectively, while the carrier concentration of *p*- and *n*-type doped porous Si layer are increased to 4.6 × 10^19^ and 2.3 × 10^19^ cm^−3^ after SOD doping. As the reference, the carrier concentration of *p*- and *n*-type Si substrate are 2.3 × 10^19^ and 9.0 × 10^18^ cm^−3^, respectively. The Seebeck coefficient of Si substrate with doped porous Si samples decreases due to the increasing of carrier concentration caused by doped porous Si layer.

**Fig. 5:**
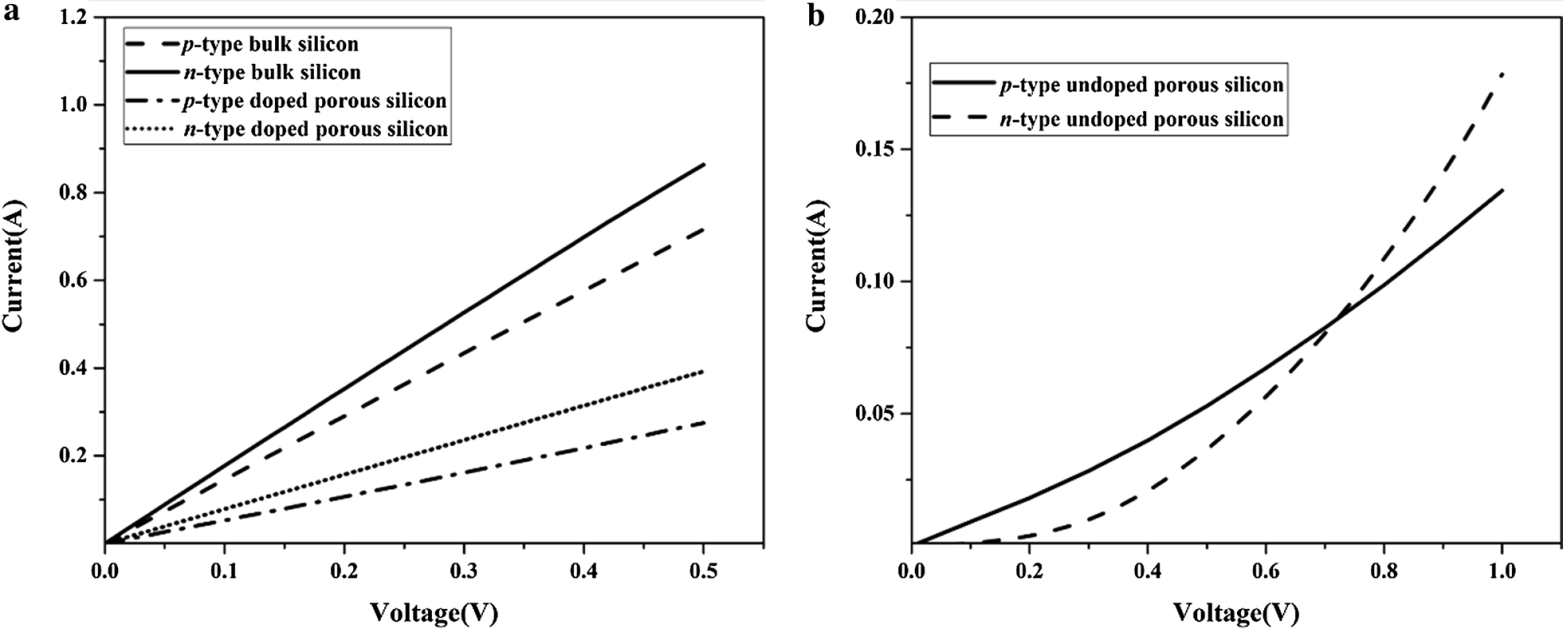
Evaluated *I*–*V* characteristics of the test specimens. **a** *p*- and *n*-type bulk Si and Si substrate with doped porous Si. **b** *p*- and *n*-type Si substrate with undoped porous Si

Figure 5a depicts the *I*–*V* curves of *p*- and *n*-type bulk Si layers along the wafer thickness direction. The total resistances of *p*- and *n*-type bulk Si layers are 1.12 and 0.65 Ω while the estimated interior resistance of *p*- and *n*-type bulk Si is both only 0.08 Ω; thus, the total resistances of *p*- and *n*-type bulk Si layers are mainly determined by contact resistance. Since *p*- and *n*-type bulk Si substrates are both highly doped (~ 10^19^), the electrical contact between the Al pad and Si substrate would be ohmic contact. However, *I*–*V* characteristics of *p*- and *n*-type Si substrates with undoped porous Si layers exhibit a nonlinear curve, as shown in Fig. 5b. In this case, we defined the electrical conductivity of these samples from the gradient of *I*–*V* curve at particular voltage of 1 V. One of the reasons for this nonlinear behavior is that the surface-to-volume ratio increases in nanostructures and a large number of surface energy states are formed on the surface of undoped porous Si layers, thus the nonlinearity is caused by quantum confinement effect [34]. Unlike bulk Si, the electrons are required to have more energy to travel the Al-porous Si interface, which increases the contact resistance and decreases the total electrical conductivity. Moreover, other factors, such as metastable hydrogenated surface, and natural oxide, have effect on the electrical characteristic of porous Si, which may also contribute to the huge drops of electrical conductivity [35, 36]. After the SOD doping, ohmic contact is obtained for *p*- and *n*-type Si substrates with doped porous Si, as the linear *I*–*V* curve is shown in Fig. 5a, and the electrical conductivities of *p*-type and n-type Si substrates with doped porous Si layers increase from 150 to 1160 and 385 to 1390 S/m, respectively, as shown in Table 1. The increment of electrical conductivity mainly results from the decrease of the contact resistance because the SOD doping mainly affects the surface of porous Si. After the SOD doping, thin *p*^+^ or *n*^+^ porous Si layers are formed on *p*- and *n*-type porous Si layers, respectively, and the carrier concentration can reach to approximately 10^19^ cm^−3^. Therefore, electrons can tunnel through the interface between Al and doped porous Si regardless of potential barrier [37].

To evaluate the electrical characteristic of the test specimens, the specific contact resistance is evaluated on the basis of transmission line measurement (TLM) theory, which is defined as a contact resistance per unit area [38]. In the TLM theory, the total resistance *R* can be calculated by,

4 $$R = 2R_{{\rm{c}}} + \frac{{p_{i} }}{A}L$$

where *R*_c_ is the contact resistance, *p*_*i*_ the interior resistivity of the sample, *L* the gap between two electrodes, and *A* the cross-sectional area. The specific contact resistance *p*_*c*_ can be defined as *p*_*c*_ = *R*_*c*_ × *A*. Therefore, Eq. 4 can be converted by multiplying area *A* into,

5 $$RA = 2p_{c} + p_{i} L$$

**Fig. 6:**
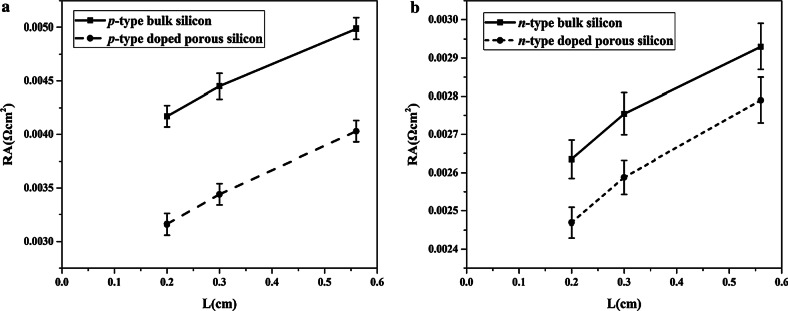
Relationship between *L* and *RA*. **a** *p*-type bulk Si and Si substrate with doped porous Si. **b** *n*-type bulk Si and Si substrate with doped porous Si

When the value *L* is approaching to 0, *R* becomes twice of the contact resistance. Thus, *p*_*c*_ can be estimated from the relationship between *RA* (total resistance multiplied by cross-sectional area) and *L*, as shown in Fig. 6. Thus, the specific contact resistance *p*_*c*_ can be obtained from the half of the extrapolated value at *L* = 0. Since the *I*–*V* curves are nonlinear, the specific contact resistances of undoped porous Si cannot be measured. The calculated specific contact resistances of *p*- and *n*- type doped porous Si were 1.35 and 1.16 mΩ-cm^2^, respectively, while *p*- and *n*- type bulk Si was 1.88 and 1.25 mΩ-cm^2^, respectively. This result shows that *p*- and *n*-type doped porous Si had lower specific contact resistance than *p*- and *n*- type bulk Si. Therefore, compared with Si substrate with undoped porous Si, lower contact resistance between doped porous Si and metal is attributed to the enhancement of electrical conductivity of Si substrate with doped porous Si.

Table 1 shows the power factor of *p*- and *n*- type bulk Si and Si substrates with undoped and doped porous Si layer. Although the Seebeck coefficient of *p*- and *n*- type Si substrates with porous Si both decreases via SOD doping, the power factor increases 4 times to 280 μW/(m K^2^) for *p*-type while it increases 2 times to 320 μW/(m K^2^) for *n*-type due to the significant increase of the electrical conductivity. In our previous research, the ZT value of Si substrates with undoped porous Si can increase 7.3 times larger than that of original bulk Si due to higher Seebeck coefficient (670 μV/K) and lower thermal conductivity (3.8 W/mK) [20]. However, further optimization of ZT value of Si substrates with undoped porous Si is impeded by relatively low electrical conductivity. Low thermal conductivity can preserve Si even by doping Si because the heat transport is mainly governed by phonons with a mean free path more than 100 nm, and thus, thermal conductivity is mainly lowered by the nanostructuring with porous Si [39]. Moreover, the best *ZT* value of material can be more than 0.1 at room temperature when the carrier concentration is ranging from 10^19^ and 10^21^ cm^−3^ based on Boltzmann transport equation [40]. Therefore, the estimated *ZT* value of Si substrates with doped porous Si can be improved to be approximately 0.1 via SOD doping, which is 5 times larger than that of Si substrate with undoped porous Si (0.02). As the consequence, Si substrates with doped porous Si via SOD doping is concluded to improve its thermoelectric performance.

## Conclusions

Porous Si was synthesized on highly doped *p*- and *n*-type bulk Si using metal-assisted chemical etching (MACE). Surface doping using spin-on-dopant (SOD) was used for improving the electrical properties of *p*- and *n*-type porous Si. Compared to the *p*- and *n*-type Si substrates with undoped porous Si (696 and 650 μV/K), the Seebeck coefficient of the *p*- and *n*-type Si substrates with doped porous Si is decreased to 491 and 480 μV/K due to the increasing carrier concentration of doped porous Si layer. Compared with the carrier concentration of *p*- and *n*-type bulk Si (2.25 × 10^19^ and 9.03 × 10^18^ cm^−3^), the carrier concentration of *p*- and *n*-type undoped porous Si is decreased to 1.3 × 10^18^ and 1.35 × 10^18^ cm^−3^ due to the quantum confinement effect and larger surface area, while the carrier concentration of *p*- and *n*-type doped porous Si is increased to 4.6 × 10^19^ and 2.29 × 10^19^ cm^−3^ after the SOD doping. However, SOD can only be used to dope thin porous Si film. Compared with *p*- and *n*-type undoped porous Si, *p*- and *n*-type doped porous Si increased the electrical conductivity from 150 to 1160 and 385 to 1390 S/m due to the decreasing of contact resistance. Moreover, the ohmic contact can be obtained in *p*- and *n*-type doped porous Si. The special contact resistance between porous Si and Al is decreased to 1.35 and 1.16 mΩ-cm^2^, which is lower than the contact resistance between bulk Si and Al due to the increasing of carrier concentration. Even though the Seebeck coefficient decreases, the power factor of *p*- and *n*-type Si substrate with doped porous Si is increased to 280 and 320 μW/(m·K^2^), respectively, due to the enhancement of the electrical conductivity. Therefore, Si substrate with porous Si formed by MACE after SOD doping increases the electrical conductivity and can improve the thermoelectric performance of porous Si, which is expected to employ for thermoelectrical application.

## Data Availability

The datasets used and/or analyzed during the current study are available from the corresponding author on reasonable request.

## Abbreviations

Si: Silicon
MACE: Metal-assisted chemical etching
SOD: Spin on dopant
FE-SEM: Field emission scanning electron microscopes
RIE: Deep reactive ion etching
TEOS-CVD: Tetraethoxysilane chemical vapor deposition
TLM: Transmission line measurement
